# Prevalence and associated factors of client satisfaction with family planning service among family planning users in Ethiopia: a systematic review and meta-analysis

**DOI:** 10.1186/s12905-023-02300-8

**Published:** 2023-03-30

**Authors:** Temesgen Geta, Nefsu Awoke, Tadele Lankrew, Eshetu Elfios, Eskinder Israel

**Affiliations:** 1 School of Nursing, Wolaita Sodo University, Wolaita, Ethiopia; 2 School of Public Health, Wolaita Sodo University, Wolaita, Ethiopia

**Keywords:** Client satisfaction, Family planning service, Women, Ethiopia, Systematic review, Meta-analysis

## Abstract

**Background:**

Considering the importance of client satisfaction in the quality of family planning services, a regular evaluation should be carried out. Several studies have been conducted in Ethiopia, but so far there were no pooled estimates of the prevalence of customer satisfaction with family planning services in the country. Therefore, this systematic review and meta-analysis was intended to estimate the pooled prevalence of client satisfaction with Ethiopian family planning services in Ethiopia. The findings of the review can be used to develop strategies and draft policies in the country.

**Methods and materials:**

This review included articles published only in Ethiopia. The main databases were Medline/PubMed, Web of Science, Google Scholar, Scopus, Ethiopian University Repository Online, and Cochrane Library. Cross-sectional studies conducted in English and meeting the eligibility criteria were included in the review. A random-effects meta-analysis was performed. Data extraction and analysis were performed using Microsoft Excel and STATA version 14 software, respectively.

**Result:**

The pooled prevalence of customer satisfaction with family planning services in Ethiopia was 56.78% [(95% CI (49.99, 63.56); I^2^ = 96.2%, p < 0.001]. Waiting time > 30 min [OR = 0.2, 95% CI (0.1–0.29), I^2^ = 75.0%, *p* < 0.001], privacy maintained [OR = 5.46, 95% CI (1.43–20.9), I^2^ = 95.8%, *p* < 0.001], education status [OR = 0.47], 95% CI (0.22–0.98), I^2^ = 87.4%, *p* < 0.001] was significant in client satisfaction related to family planning services.

**Conclusion:**

According to this review, client satisfaction with family planning services in Ethiopia was 56.78%. In addition, waiting time, women's educational level, and respect for privacy were identified as factors that both positively and negatively impact women's satisfaction with family planning services. Decisive action, such as educational intervention, continued monitoring and evaluation of family planning services, and arranging training for providers, is required to address identified issues and ensure higher levels of family satisfaction and utilization. This finding is important for shaping strategic policies and improving the quality of family planning services. This finding is important for designing strategic policy and increasing the quality of family planning services.

## Background

According to the World Health Organization (WHO), family planning (FP) is the ability of an individual or couple to determine the number of children, space for the children, and timing of their children [1]. It helps couples meet their reproductive health goals and enables them to exercise their right to have children. It promotes maternal and baby health by improving health-related outcomes, such as reducing unwanted and high-risk pregnancies, maternal and neonatal mortality, the likelihood of disease transmission, and unsafe abortions [2–4].

Beyond the individual level, FP benefits at the population level. It is an important strategy that will play a key role in curbing rapid population growth and achieving the 2030 Sustainable Development Goals. In order to achieve these goals and a high utilization of FP, we need to improve overall client satisfaction with our FP services [2, 3]. Customer satisfaction (CS) with FP is one of the main factors influencing the use of FP and all other reproductive health services [5, 6]. Scientific evidence from developing countries shows that dissatisfaction with FP services is a leading cause of contraceptive discontinuation, unwanted pregnancies, high birth rates, and non-compliance [3, 7].

Client satisfaction refers to patient value judgments and subsequent reactions to what they perceive in a healthcare facility immediately before, during, and after a clinical visit. It is the customer's assessment of what they want and expect from their healthcare service. Collecting the right information on customer satisfaction is a key factor in helping healthcare facilities make critical decisions related to operational and care planning. Evidence from Africa found that client satisfaction with family planning was significantly higher in private health centers. Variation was influenced by factors such as shorter waiting times and adequate availability of contraceptive methods and supplies [8, 9].

Many authors in low- and middle-income countries use CS as an indicator to assess the quality of FP and other health services and to identify influencing factors related to quality of care in family planning services [9]. Satisfying family planning users by providing clinically effective and responsive care is critical to increasing contraceptive use. This includes respectful, safe, and trustworthy interactions with users, emotional support, satisfaction with family planning services provided, and comprehensive educational counseling [10, 11].

Previous studies have shown that CS status by FP services is significantly influenced by various factors such as privacy, side effects explained to the client, distance to medical facilities, waiting time, education level, age, and marital status of the woman [5, 7–12]. Reviews from different countries indicate that the prevalence of CS is 99% in Tanzania [7], 86% in Mozambique [5], 80% in Mexico [13], 89% in Nepal [14], 41.7% in Jigjiga [15], 75.3% in Hosanna [10], and 66.1% in Bahirdar [16].

Despite high client satisfaction with FP services in certain regions, the 2016 Ethiopian Demographic and Health Survey (EDHS) reported that over a third of women had stopped using FP within a year [17]. To address this issue, the Ethiopian government has launched a health transformation program aimed at increasing FP use to 55% by 2020 and minimizing the unmet need for FP use to 10% but, progress has been slow since mini-EDHS (2019) found the contraceptive rate to be 41% [18, 19]. The WHO recommends that improving client satisfaction is the most important strategy for improving the quality and effectiveness of healthcare service delivery, including FP. Similarly, providing FP services alone does not improve their use and promote the health of mothers and babies. Therefore, great attention should be paid to client satisfaction and related factors for her FP services in developing countries like Ethiopia [20].

Studies have been conducted to assess customer satisfaction and related factors with FP services in Ethiopia, but the representativeness and results of any single study are neither conclusive nor consistent. Moreover, the status of CS with FP at the national level remains unclear. Therefore, the aim of this systematic review and meta-analysis was to determine the prevalence of client satisfaction with FP in Ethiopia and to identify factors influencing women's satisfaction with FP services. The results of this study lead to general findings that help improve the quality of contraceptive services by helping to develop policies, design strategies, and improve the use of FP. It plays an important role in reducing maternal and neonatal mortality.

## Methods and materials

A systematic review and meta-analysis were performed to estimate the overall prevalence and associated factors of CS with FP services in Ethiopia.

### Search strategy

Studies included in this study were searched using Medline/PubMed, Web of Science, Google Scholar, Scopus, the Ethiopian University Repository online, and the Cochrane Library. In addition, missing data were handled by contacting the corresponding authors. Additionally, missing data were processed by contacting the corresponding authors. Through the standard Population Intervention Comparisons and Outcomes (PICO) questions, a comprehensive search strategy was developed using various Boolean operators. The following search terms were used: "or" and "and". Using Boolean operators, satisfaction and 'family planning or contraception' or client or woman or mother and related factors and Ethiopia and related terms (Amhara, Oromia, Somalia, Southern National Ethnic Region (SNNPR), Tigray, Sidama, Gambella, Afar, Benshangul regions, and Addis Ababa and Dire Dawa city adminstrarion). All studies retrieved from the database were exported to the Endnote library and searched first by title and abstract. The full text of these articles met the selection criteria for title and abstract and was read in full. A systematic review with narrative synthesis was used to estimate the outcome of the articles in Ethiopia. For homogeneous articles, a meta-analysis was considered.

### Eligibility criteria

#### Inclusion and exclusion criteria

The articles included in this review assessed client satisfaction with FP services and related factors among Ethiopian FP service users. In addition, this review included studies published in English that were conducted using a cross-sectional study design. This review excluded studies conducted outside of Ethiopia and study designs other than cross-sectional studies.

### Data extraction and quality assessment

Data extraction was based on parameters such as author name, year of publication, a place where the studies were conducted, the sample size of each study, and the study design. We (TG, EE, and EI) used Microsoft Excel spreadsheets to collect key data from the accepted articles. The authors (TG, NA, and TL) independently extracted data from those included. After consensus and detailed discussion on data extraction was reached and significant analyzes were performed using the Joanna Brings Institute Meta-Analysis of Statistics Assessment and Review Instrument (JBI-MASTER), studies meeting the inclusion criteria were included and tabulated. Articles are checked for quality before selection for final review. Studies with a quality indicator score of 7 or higher on her were classified as low risk. (Table 1).

**Table 1:** Critical appraisal results of eligible studies in this study on client satisfaction with family planning services and associated factors among family planning users in Ethiopia, 2022

Name of author	Q1	Q2	Q3	Q4	Q5	Q6	Q7	Q8	Q9	Total
Baruda. F, et al. [21]	Y	Y	Y	Y	Y	Y	Y	Y	Y	9
Tadergew.MM [22]	Y	Y	Y	N	Y	Y	Y	Y	Y	8
Dulla. A, et al. [23]	Y	Y	Y	Y	Y	Y	N	Y	Y	8
Argago et al. [10]	Y	Y	Y	Y	Y	Y	Y	Y	Y	9
Wogu. et al. [24]	Y	Y	Y	Y	Y	Y	Y	Y	Y	9
Tsegaye Beyene. et al. [25]	Y	Y	Y	Y	Y	Y	Y	Y	Y	9
Ayano Wakjira B [12]	Y	Y	Y	Y	Y	Y	Y	Y	N	8
Desalign et al. [26]	Y	Y	Y	U	Y	Y	Y	Y	Y	8
Bezawit. B, et al. [27]	Y	Y	N	Y	Y	Y	Y	Y	Y	8
Teshale. G, et al. [28]	Y	Y	Y	Y	Y	Y	Y	Y	Y	9
Asrat, et al. [16]	Y	Y	Y	Y	Y	Y	Y	Y	Y	9
Gebreyesus [15]	Y	Y	Y	Y	Y	Y	Y	Y	Y	9

*Y* Yes, *N* No, *U* Unclear, JBI critical appraisal checklist for studies reporting prevalence data: Q1 = was the sample frame appropriate to address the target population? Q2-Were study participants sampled appropriately? Q3-Was the sample size adequate? Q4-Were the study subjects and the setting described in detail? Q5-Was the data analysis conducted with sufficient coverage of the identified sample. Q6-Were the valid methods used for the identification of the condition? Q7-Was the condition measured in a standard, reliable way for all participants? Q8-Was there appropriate statistical analysis? Q9-Was the response rate adequate, and if not, was the low response rate managed appropriately?

### Data processing and analysis

Data were extracted using a Microsoft Excel spreadsheet and the extracted data were analyzed using STATA version 14. Using a random-effects model meta-analysis, we calculated the overall CS including factors associated with his FP service in Ethiopia. Publication bias was checked by visual assessment using funnel plots. Cochrane Q-Static and I^2^ were used to confirm heterogeneity between studies. Subgroup analyzes were performed to compare the pooled prevalence of CS with FP and related factors across regions. Pooled prevalence was presented in forest pilot format with 95% CI.

### Identification and characteristics of the included studies

**Fig. 1:**
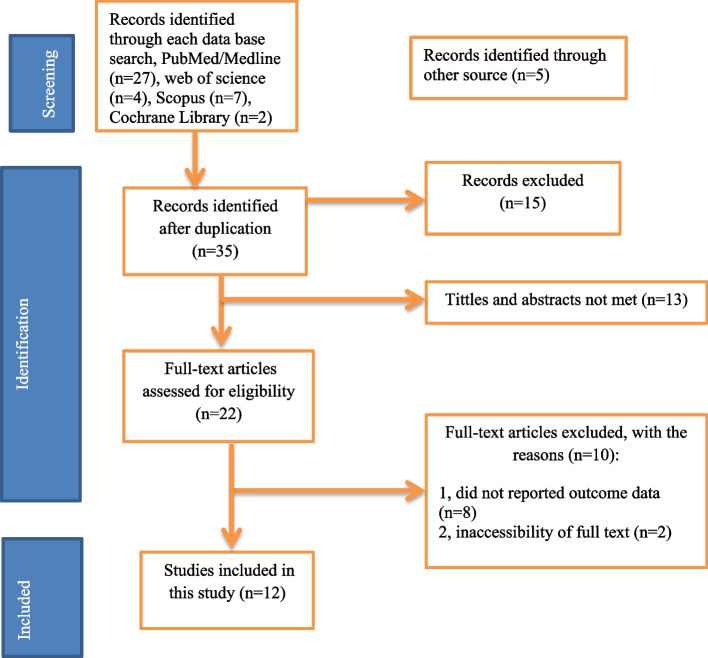
Flow chart of study selection for systematic review and meta-analysis of CS with FP and its associated factors among FP users in Ethiopia, 2022

From November to December 2022, more than 50 articles were identified in major electronic databases and additional applicable sources were searched. Of these identified items, 15 articles were removed for duplication and 35 articles were reserved for further review. Thirteen studies were excluded because their abstracts and titles did not meet the requirements. Of the remaining 22 articles, 10 studies were excluded because they conflicted with the inclusion criteria set for that study. Finally, 12 studies that met the eligibility criteria were included in this study. (Fig. 1).

A total of 12 articles with 5087 participants were included in this systematic review and meta-analysis. All included studies were cross-sectional studies and sample sizes ranged from [21] to 604 [26]. Regarding the regional distribution of the included studies, four were from the Oromia region [12, 25–27], two from the Amhara region [16, 28], one from Somalia [15], and five from Southern Nations, Nationalities, and Peoples’ Region (SNNPR) [10, 21–24] (Table 2).

**Table 2:** Study characteristics included in the systematic review and meta-analysis

Name of author	Year	Region	Study area	Study Design	S.S	Cases	Prevalence
Baruda. F et al. [21]	2018	SNNPR	Gurage	Cross-sectional	416	278	67%
Tadergew. M et al. [22]	2021	SNNPR	Halaba	Cross-sectional	370	263	71.2%
Dulla. A et al. [23]	2019	SNNPR	Gofa	Cross-sectional	538	368	68.4%
Argago et al. [10]	2015	SNNPR	Hosana	Cross-sectional	324	244	75.3%
Wogu. et al. [24]	2020	SNNPR	Tembaro	Cross-sectional	407	187	46%
Tsegaye Beyene. et al. [25]	2022	Oromia	Adama	Cross-sectional	417	217	52%
Ayano Wakjira B et al. [12]	2016	Oromia	Wonji	Cross-sectional	300	135	45%
Desalign. T et al. [26]	2020	Oromia	Jimma town	Cross-sectional	604	258	42.8%
Bezawit. B, et al. [27]	2019	Oromia	Jimma zone	Cross-sectional	278	128	46%
Teshale. G, et al. [28]	2022	Amhara	Gonder	Cross-sectional	477	257	53.9%
Asrat, et al. [16]	2018	Amhara	Bahirdar	Cross-sectional	490	324	66.1%
Gebreyesus [15]	2019	Somali	Jigjiga	Cross-sectional	492	232	47.1%

### Prevalence of client satisfaction with FP services in the Ethiopia

**Fig. 2:**
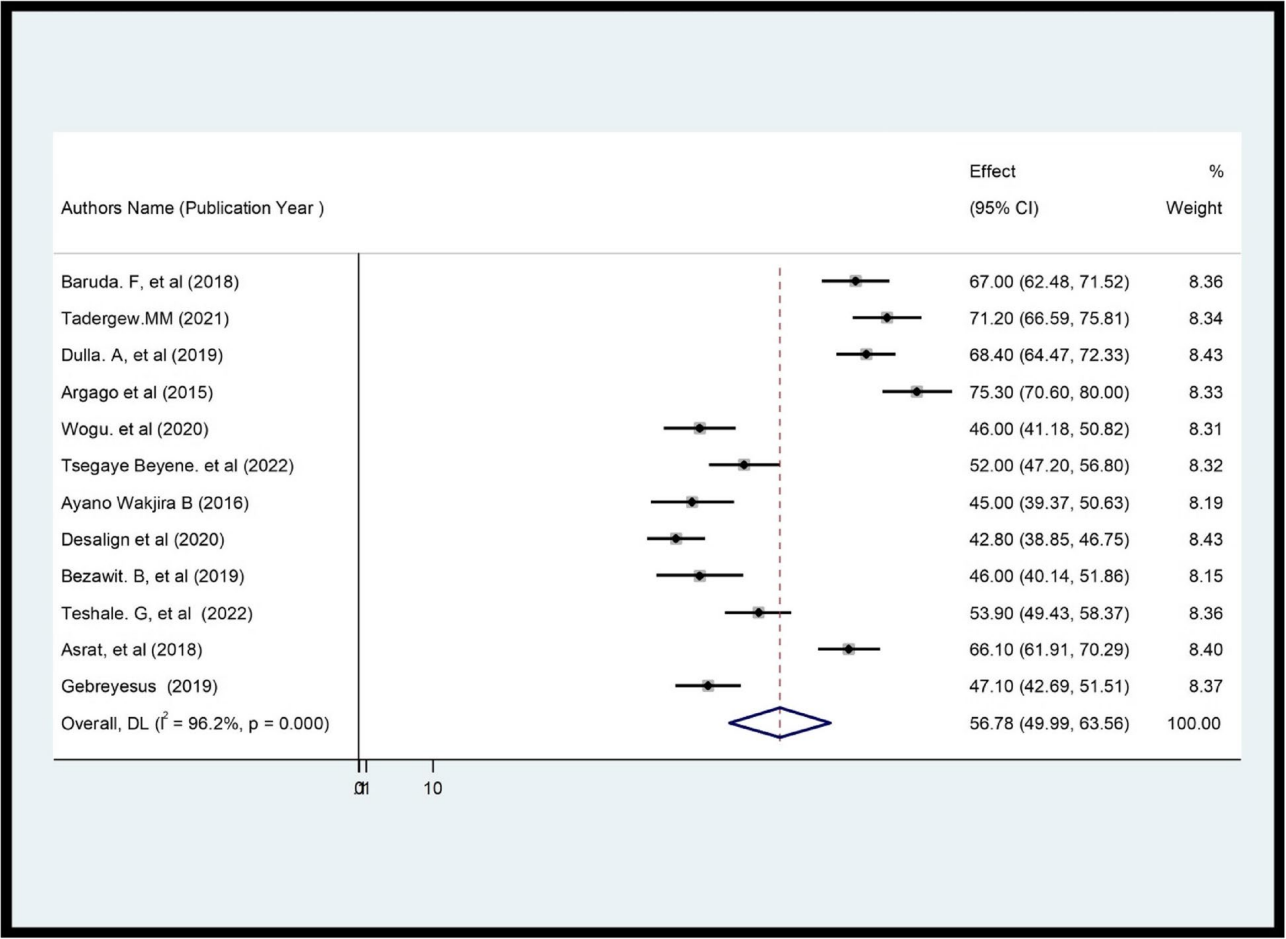
Forest plot showing the pooled prevalence of CS with FP service in Ethiopia (*n* = 5087)

Among the included studies, the prevalence of client satisfaction with family planning ranged from 42.8 [26] to 75.3 [10]. The pooled estimated prevalence of CS with FP treatment among Ethiopian contraceptive users was 56.78% [95% CI (49.99; 63.56); I^2^ = 96.2%, *P* ≤ 0.001] (Fig. 2).

### Subgroup analysis of CS with FP service in Ethiopia

**Fig. 3:**
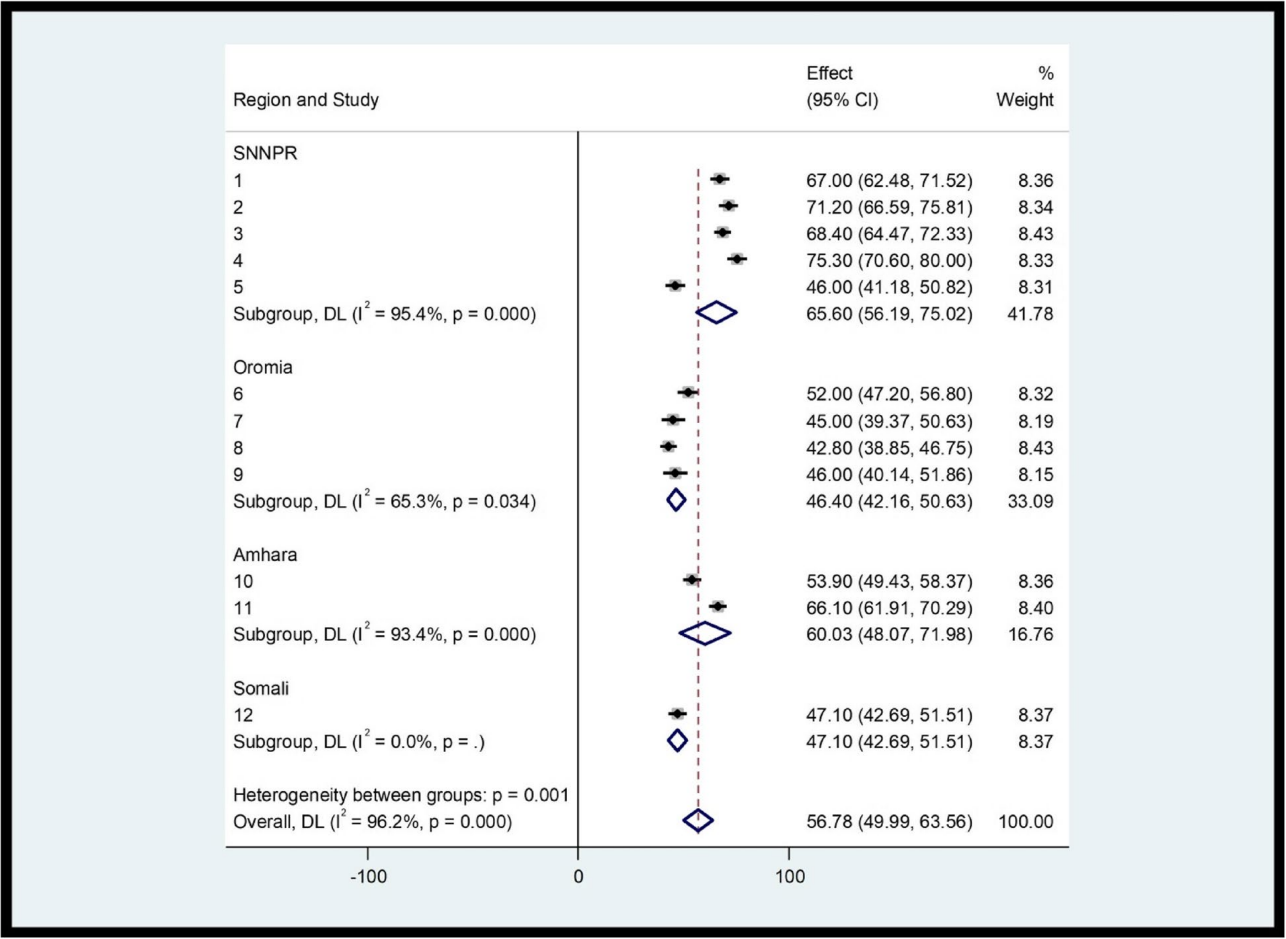
Subgroup analysis of CS with FP among FP users by region in Ethiopia (*n* = 5087)

In subgroup analyzes performed in each region, the highest prevalence of CS was observed in SNNPR with values ​​of 65.6% (95% CI: 56.9, 75.2) and Amhara with 60.3% (48.07; 71.9), followed by the Somali region 47.1% (46.69, 51.51). The lowest values ​​were observed in the Oromia region, at 46.4 (42.16, 50.63) (Fig. 3).

### Hetrogenicity and publication bias

**Fig. 4:**
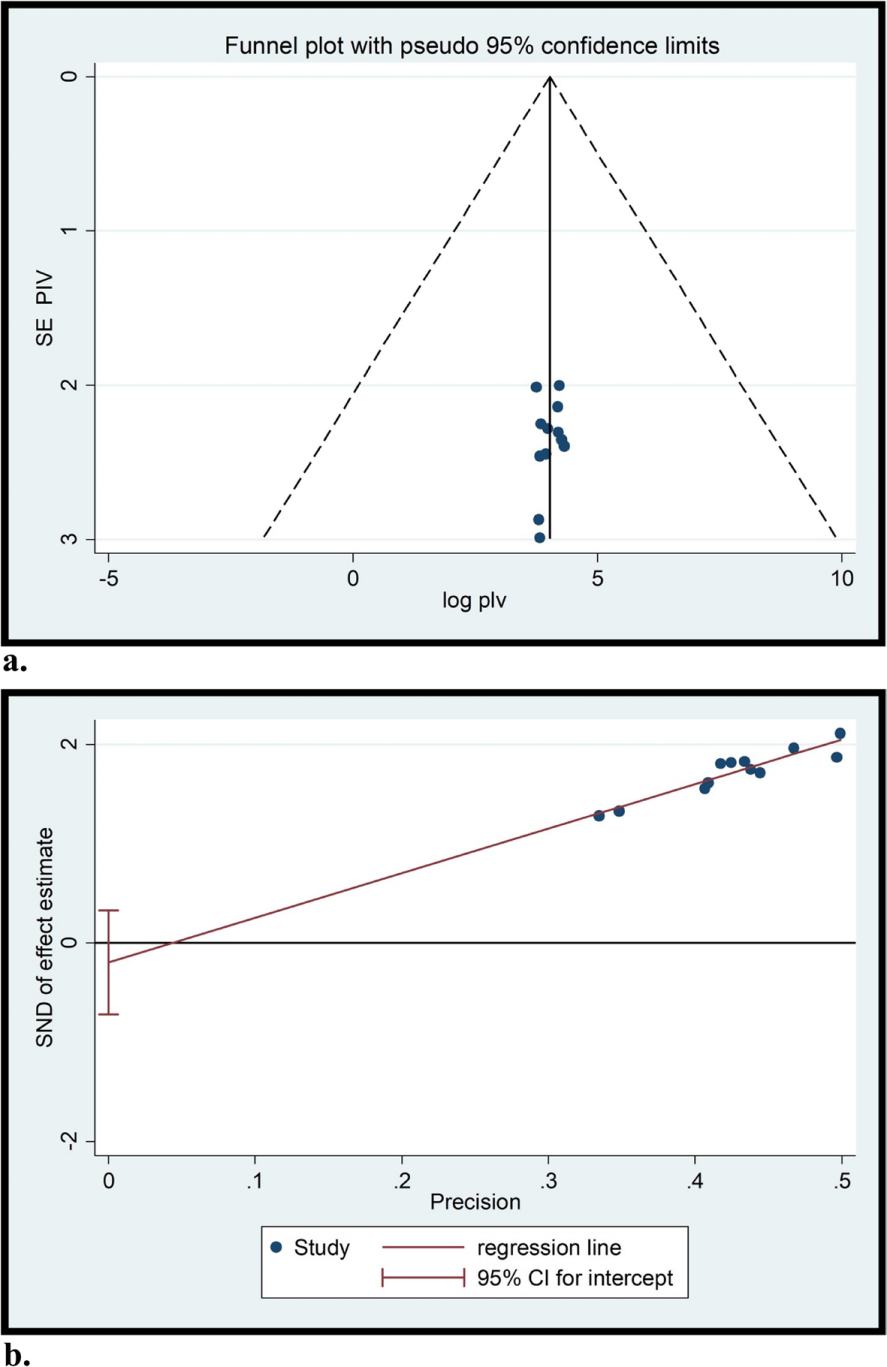
(**a**) funnel plot and (**b**) Eggers test of the study

To minimize and balance study heterogeneity, subgroup analyzes were performed by region. The results of the I^2^ test indicate that there was considerable heterogeneity between studies (I^2^ = 96.2%, *p*-value of *P* ≤ 0.001) (Fig. 2). In addition, a random-effects model was used to control for study heterogeneity. Publication bias of the studies was monitored by Egger's test and visual inspection of the funnel plots. Funnel plot results showed that the selected studies had a symmetrical distribution after inspection (Fig. 4a) and Egger's test (*P* = 0.420) (Fig. 4b). This indicates no publication bias.

### Associated factors with client satisfaction with family planning

**Fig. 5:**
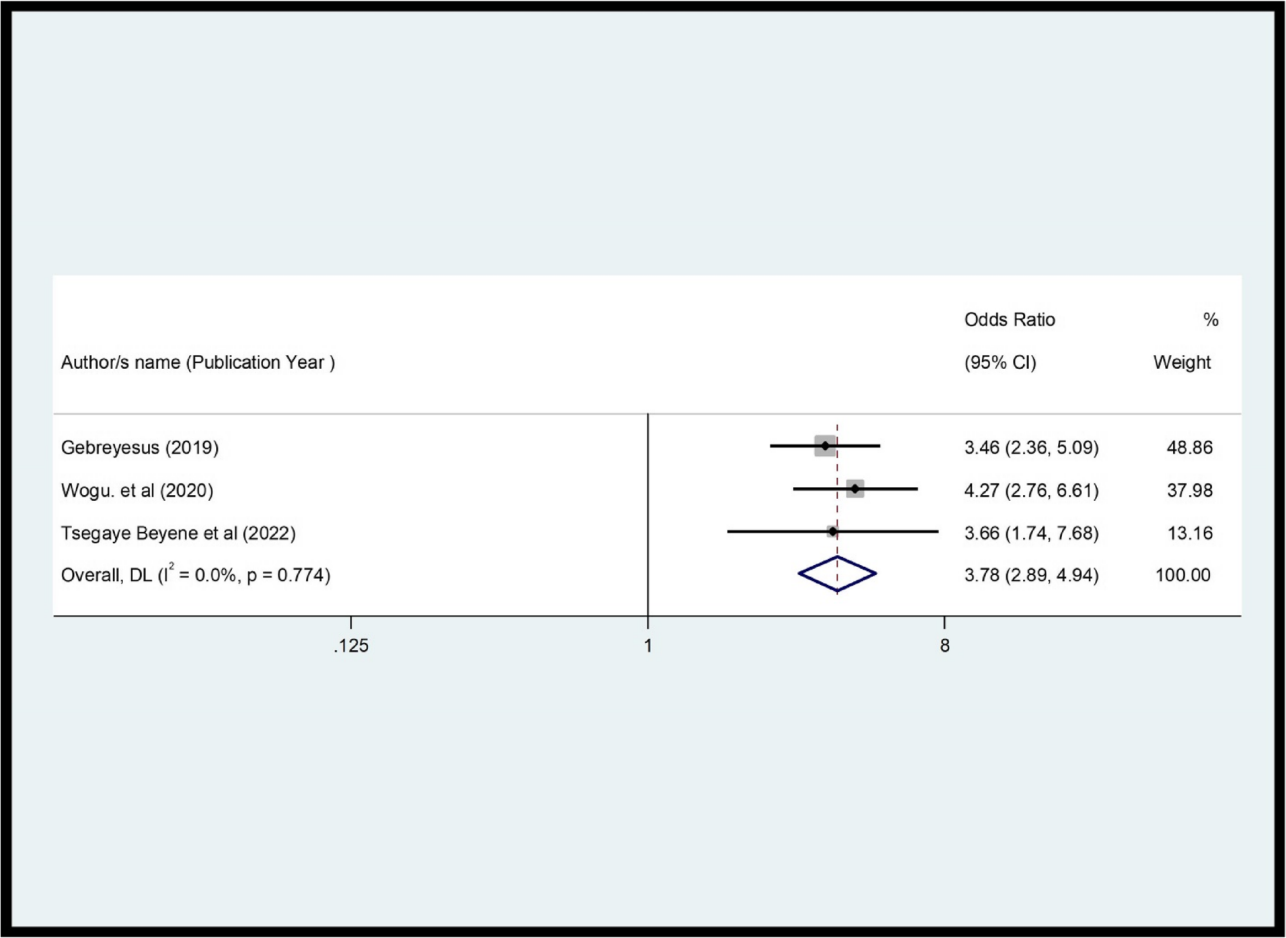
Pooled OR of the association between side effects explained and CS among FP users in Ethiopia, 2022

In this review, three variables ( privacy maintained, educational level, and waiting time) were significantly associated with FP service, while the side effects explained were not significantly associated with CS (*p* = 0.774) (Fig. 5).

**Fig. 6:**
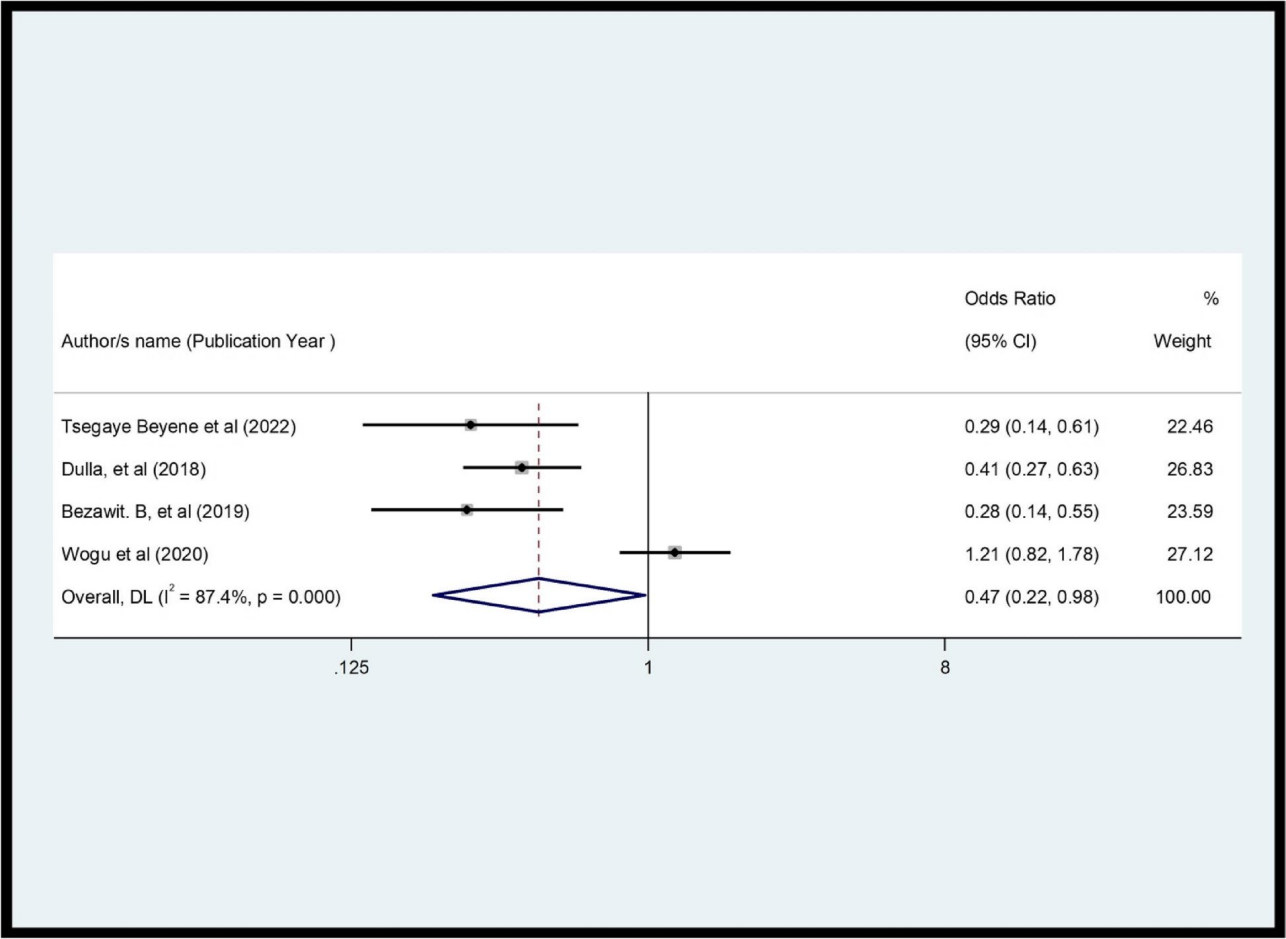
Pooled effect (OR) of the association between educational level and CS among FP users in Ethiopia, 2022

**Fig. 7:**
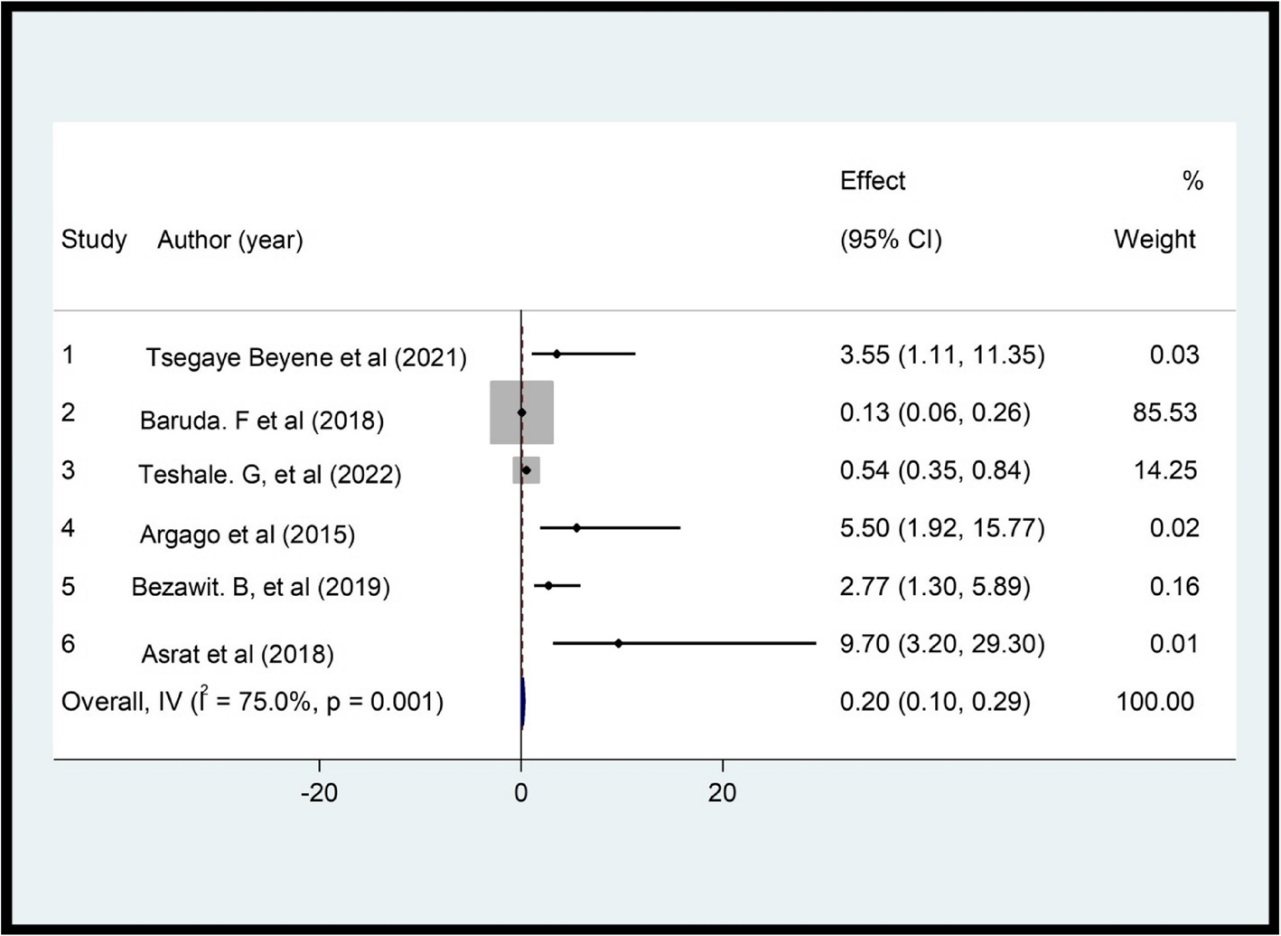
Pooled OR of the association between waiting time and CS among FP users in Ethiopia, 2022

**Fig. 8:**
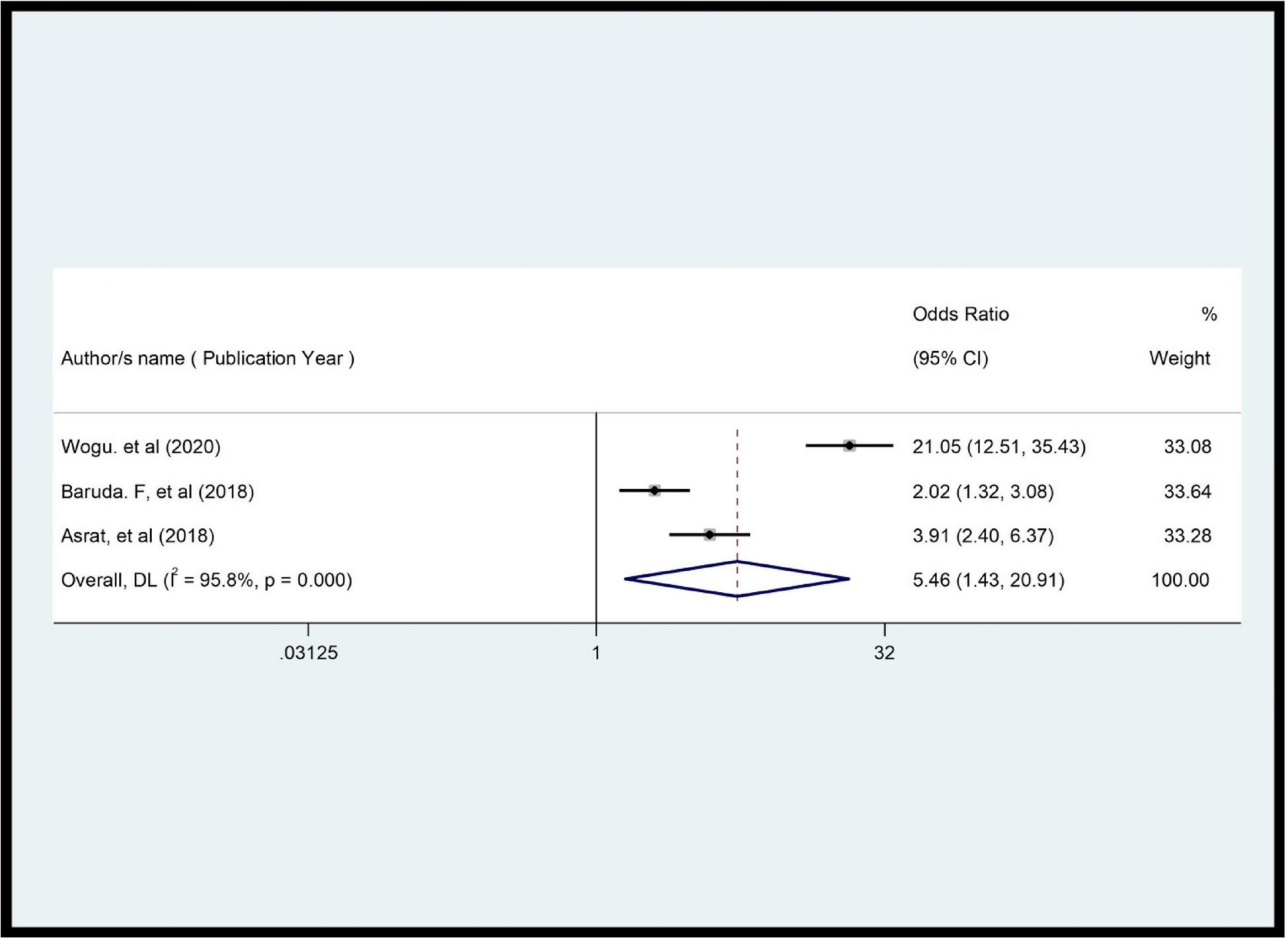
Pooled OR of the association between privacy maintained and CS among FP users in Ethiopia, 2022

The review showed a significant association between women's level of education and CS. Women with higher education had 53% higher satisfaction than those with lower education (OR = 0.47, *p* = 0.001, I^2^ = 87.4%) (Fig. 6). This review demonstrated that there was a significant association between waiting time and CS (OR = 0.2, *p* = 0.0001, I^2^ = 75.0%) (Fig. 7). In this review, women whose privacy was maintained were 5.46 times more likely to be satisfied with family planning services compared to their contrast group (OR = 5.46, *p* = 0.000, I^2^ = 95.8%) (Fig. 8).

## Discussion

In this systematic review and meta-analysis, the pooled estimate of women's satisfaction with existing FP provisions in Ethiopia was 56.78% [95% CI: 49.99 to 63.56; I2 = 96.2%]. The results of this study were consistent with studies in Bangladesh (62%)[29] and Ghana (59.3%) [3]. Studies done in Kenya (81%) [30], Nigeria (85%) [6], Tanzania (86%) [31], Mozambique (91%) [5], and Portal Said City (95.4%) [32] showed higher results. Possible reasons for the discrepancies could be due to differences in study participant characteristics, quality of family planning services, policies and strategies, research areas, sampling methods, and participant sample size. In addition, measurement tools that quantify CS levels and low job satisfaction among health care providers contribute to low customer satisfaction in Ethiopia.

Regarding subgroup analyses, the highest prevalence of CS with FP was observed in SNNPR, with the lowest values ​​in the Oromia region. Possible reasons for this discrepancy are differences in standards of care, differences in compassionate and respectful maternal care practices, or provider experiences.

In this meta-analysis, participants' education level was significantly associated with client satisfaction. This result was consistent with a single study conducted in Ghana, Tanzania, and Kenya [3]. This is because when there is a higher educational level, client expectations and cooperativeness could be higher, which increases the clients’ satisfaction status [24]. Waiting times of 30 min or longer could have a significant impact on client satisfaction. Women who waited more than 30 min for service were less satisfied than women who waited less than 30 min. This study is consistent with the studies carried out in Nigeria [6], Mozambique [5], and Ethiopia [16, 21, 24, 26]. A possible reason is that Ethiopia has a limited number of service providers and thus specific working hours, which may lead to longer stays for FP recipients. Reducing wait times increases client satisfaction and improves the quality of family planning services.

This review found that women whose privacy was maintained during service delivery were 5.46 times more likely to be satisfied with FP services than women whose privacy was not maintained during service delivery. This means that health institutions and their providers of services must play an important role in protecting privacy while providing services. This is because family planning is a very personal subject, and people do not like to openly discuss their problems. So, ensuring privacy, particularly during the examination and counselling time, is essentail in offering family planning services. This finding was supported by previous studies [10, 16, 23].

## Conclusion

The review found that client satisfaction with family planning services in Ethiopia is relatively low (56.78%). This indicates that about 43% of the study participants were dissatisfied with FP services in Ethiopia. The Ethiopian government particulrly Ministry of Health should take the following urgent measures: Organization of training for all providers, regular monitoring and evaluation, and supervision of FP services provided by health care professionals.

In addition, women education level, waiting time, and privacy were factors related to client satisfaction with family planning services. Therefore, healthcare providers are strongly encouraged to consider women's level of education when providing her FP services, reduce waiting times during service delivery, and maintain client privacy. This review has certain limitations. The included studies were dissimilar in quality as observed by heterogeneity. Most of the included studies were restricted to specific regions (southern Ethiopia, Oromia, and Amhara).

## Data Availability

The datasets generated and/or analyzed during the current study are not publicly available for because of to prevent any kinds of misuse by the public before publication but are available from the corresponding author upon reasonable request.
